# The Variation of Riverbed Material due to Tropical Storms in Shi-Wen River, Taiwan

**DOI:** 10.1155/2014/580936

**Published:** 2014-01-12

**Authors:** Chin-Ping Lin, Yu-Min Wang, Samkele S. Tfwala, Ching-Nuo Chen

**Affiliations:** Department of Civil Engineering, National Pingtung University of Science and Technology, Pingtung 91201, Taiwan

## Abstract

Taiwan, because of its location, is a flood prone region and is characterised by typhoons which brings about two-thirds to three quarters of the annual rainfall amount. Consequently, enormous flows result in rivers and entrain some fractions of the grains that constitute the riverbed. Hence, the purpose of the study is to quantify the impacts of these enormous flows on the distribution of grain size in riverbeds. The characteristics of riverbed material prior to and after the typhoon season are compared in Shi-Wen River located at southern Taiwan. These include grain size variation, bimodality, and roughness coefficient. A decrease (65%) and increase (50%) in geometric mean size of grains were observed for subsurface and surface bed material, respectively. Geometric standard deviation decreased in all sites after typhoon. Subsurface material was bimodal prior to typhoons and polymodal after. For surface material, modal class is in the gravel class, while after typhoons it shifts towards cobble class. The reduction in geometric mean resulted to a decrease in roughness coefficient by up to 30%. Finally, the relationship of Shields and Froude numbers are studied and a change in the bed form to antidunes and transition form is observed, respectively.

## 1. Introduction 

Fluvial processes are not uniform through time. Often, storm events occur during which the rate of fluvial process is highly accelerated. In these events, polishing, grinding, and breaking of the transported material take place [1]. Taiwan is located in a subtropic area and as a result it is flood prone and is characterised by typhoons and torrential rains, which brings about two-thirds to three-quarters of the annual rainfall amount. These typhoons and torrential rains are frequent during summer-autumn season (June to August) and have a mean precipitation of 2500 mm/year and reach 3000–5000 mm/year in the mountainous regions. Consequently, the capacity of rivers to mobilise sediments increases. The flood does not only erode riverbeds and coasts and damage river structures but also affects the geomorphology and topography of rivers. Due to the effects of geography and typhoons, most of the rivers in Taiwan are intermittent rivers with steep slopes, great disparity in discharge, and high sediment transport rates [2, 3]. Scour and fill of the riverbed in a steep slope intermittent river is quite different from that in a mild slope perennial river as illustrated by [4] in their investigation of typhoon induced scours in Taiwan rivers.

The nature of riverbed has a significant influence on the river's morphological and hydrodynamic characteristics. Riverbeds typically exist in two forms: gravel and sand beds. Sand beds are comprised of sand and smaller sized particles. Gravel beds consist of a wide range of particles, from fine sediments to gravel or coarser particles. The gravel streambeds are vertically stratified in three layers, in terms of particle size [5]. The top two layers of the streambed, the pavement and subpavement, are the most significant in determining the hydraulic characteristics of the stream. How the riverbed grain size is distributed is one of the most important factors that influence a stream's hydraulic, geomorphologic, and ecological health. This distribution, which in the case of Taiwan will be greatly influenced by Typhoons, affects streambed stability, sediment transport rates, and flood levels by defining the roughness of the stream channel. Roughness of the stream channel will determine frictional resistance. This resistance of the bed to flow affects the average flow velocity in a cross section for a given discharge, hence, affecting the energy the flow has for sediment transport. Consequently, it will play a significant role in the total supply and type of riverbed materials supplied. Reference [6] showed that, during exceptional events such as typhoons, boulders of up to 135 cm in diameter are transported. These observations of riverbed material have potential significance for hazard risk mitigation and stream engineering and restoration. Moreover, the riverbed analysis may bring significant information regarding solid flow, sediment sources, and their behaviour at riverbed deposits fluvial processing. References [7, 8] illustrated the role of grain size variability on sediment transport. Reference [9] investigated the sediment mobility in a large gravel-bed and demonstrated the relationship between shear stress and sediment transformation.

Studies of changes in riverbed size distributions along river channels have focused on the tendency of downstream fining [1, 10, 11]. However, studies on variability of riverbed material, especially due to typhoons and heavy torrential rains, are limited due to the difficulties associated with sampling of riverbed material, such as time consuming and costly field work. Reference [12] states that volumetric sampling, which is one of the most commonly used methods, is a significant obstacle for those that study the phenomena. They further point out that the sample in situ measurement would be more than a tonne implying extraordinary effort for those doing the sampling. Regardless of the challenges outlined, precise information on riverbed material is still required for many purposes (e.g., suspended sediment-water quality interactions, ecological and recreational problems, and river channel responses to changes in land use and rates of erosion). Hence, the objective of the paper is to assess the changes in riverbed induced by typhoons and torrential events in Shi-Wen River.

## 2. Materials and Methods

### 2.1. Study Area

**Figure 1:**
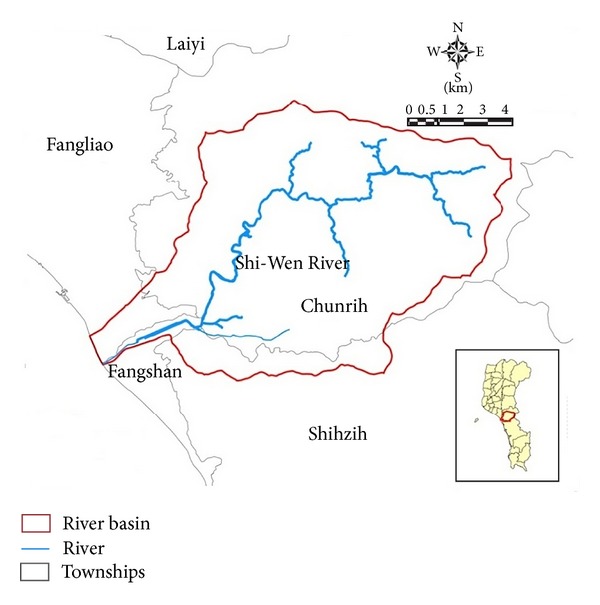
A sketch of Shi-Wen River basin.

The study was conducted at Shi-Wen River, located (21°34′48′′ North latitude and 120°47′56′′ East longitude) in the Southern part of Taiwan (Figure 1). The length of the main stream is about 22.3 km and the basin covers 89.61 km^2^. The average slope is about 0.03 with a design flood at 1300 m^3^/s (50 years return period).

**Figure 2:**
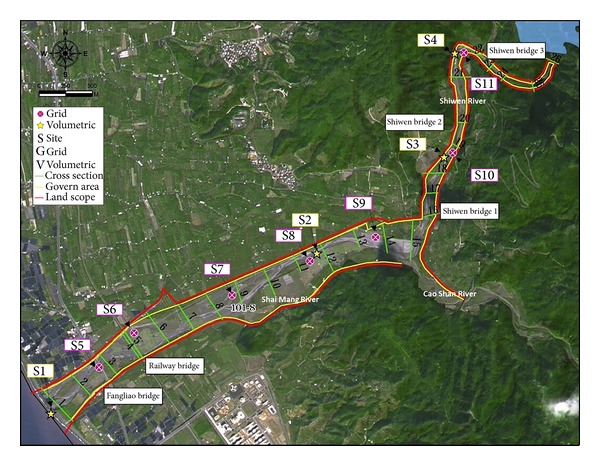
Sampling points distribution.

In general, the bed material in the studied reach is gravel; therefore, two distinct layers, surface and subsurface, are recognized. The surface layer is often called the paved or armoured layer. According to [13], rivers are considered paved if their surface and subsurface layers are composed of particles that are similar in their size, but lack sand and fine gravel in the surface layer. They are considered armoured if the particles of the surface layer are coarser than the subsurface layer. Consequently, different methods were employed to determine characteristics of these layers. In this study, we used both volumetric and grid methods to sample bed sediments before and after the typhoon season. The grid was mostly used for sampling because the procedure is simple to perform, sample is representative of the entire river reach, and the sample is reproducible [14]. However, due to its inability in representing fine particles, volumetric method was also used.  Figure 2 shows the distribution of the sampled sites (S1 to S11) and the methods used (yellow star and a pink circle, representing volumetric and grid methods, resp.). Four volumetric samples (site 1 to site 4, presented as S1 to S4 in the figure) located at a distance of about 6 km to each other were collected. The grid method was used to collect samples from seven sites (site 5 to site 11, presented as S5 to S11 in the figure) and were located at a distance of about 3 km from each other. The data obtained was put into tables from which cumulative distributions were drawn. In addition, roughness coefficients, a number of distribution parameters such as geometric mean (*D*_*g*_), geometric standard deviation (*S*_*g*_), and bimodality (*B*) index, were computed. Reference [15] proposed a *B* parameter to describe the bimodality degree. The parameter is based on the distance between two modes and on the sediment quantity obtained in the modes. The formula proposed by Wilcock is

(1) $$\begin{array}{c} \;B={\left(\frac{D_{\text{cm}}}{D_{\text{fm}}}\right)}^{0.5}\cdot \left(P_{\text{cm}}*P_{\text{fm}}\right), \end{array}$$

where *D*_cm_ is the dimension of the particles in the coarse mode expressed in mm, *D*_fm_ is the dimension of the particles in the fine mode expressed in mm, *P*_cm_ is the fraction of sediment in the coarse mode, and *P*_fm_ is the fraction of sediment in the fine mode. When *B* < 1.7, sediments are unimodal and when *B* > 1.7, sediments are bimodal. To sustain observations made on the variation of riverbed material, a Shields diagram as a function of Reynolds number and as a function of Froude number was utilized. The Shields diagram was used to observe if indeed the variation of bed material was a result of the flow conditions during typhoon season and grains moving from upstream to downstream or from outside catchment. Due to difficulties in observing flow conditions during typhoon and torrential rain events, flow data for 2-year and 10-year return period, simulated by a Hec-Ras model, was obtained from the Taiwan Water Resource Agent. The simulated flows had flows similar to the observed events in the study. Hence, the data was used to compute parameters for these diagrams.

### 2.2. Volumetric Sampling

**Figure 3:**
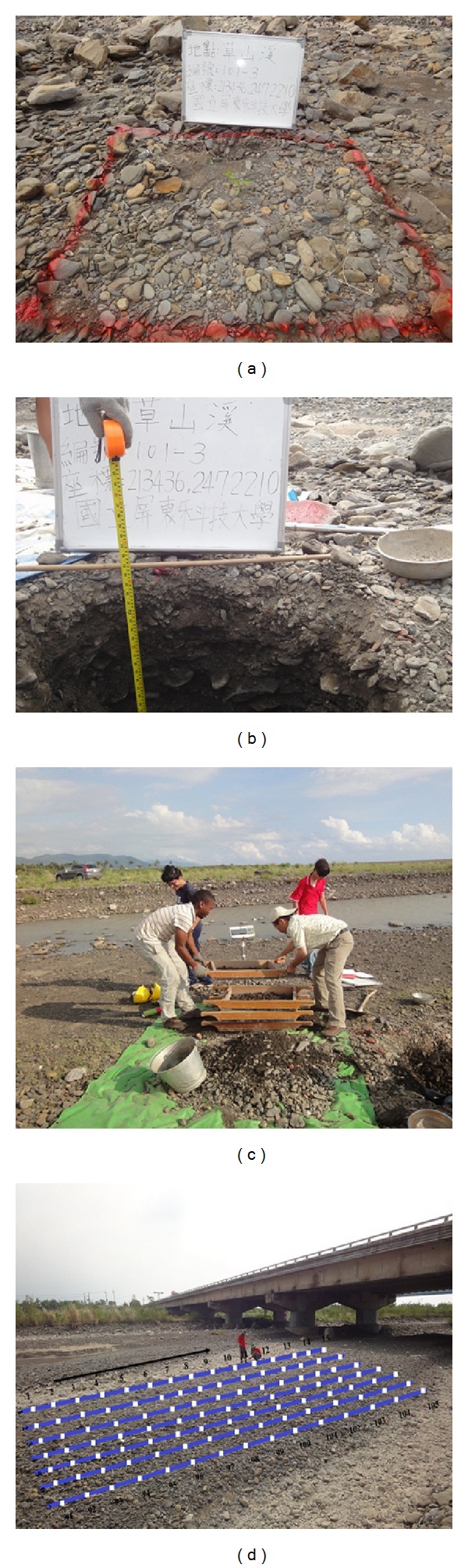
Illustration of volumetric and grid sampling methods. (a) A sampling perimeter of one square meter. (b) Vertical section of one meter. (c) Use of hand sieving to separate subsurface particles. (d) Grid method.

We identified a sampling perimeter of one square meter, sprayed with red paint (Figure 3(a)) to be a representative for each section out of which we collected subsurface samples. To measure the sediment from subsurface layer, we used a volumetric method proposed by [16]. According to this method, the total sample weight is a function of the largest clast in the sampling perimeter, which is 5% of the total mass of the sample. Some of granulometric fractions were sieved directly in the field using a set of sieves with holes having diameters according to the Wentworth scale. Four sieves were used and they featured the following hole diameters: 64 mm (−6 phi), 32 mm (−5 phi), 16 mm (−4 phi), and 8 mm (−3 phi) (Figure 3(c)). Samples with diameters between 64 mm and 128 mm were weighted and measured in the field using a special calliper. For the ones larger than 256 mm, more difficult to be weighted in the field, a diameter-weight scale conversion according to [17] was used, built on the basis of river clasts we investigated by evaluating the weight of the largest clasts on the basis of the *B* axis. Fractions smaller than 8 mm were taken to laboratory for further mechanical sieving after having been dried at 90°C in the oven. The samples that were brought to the laboratory weighed 5 kg. The sieve diameters were 4.75 mm (−2.25 phi), 2 mm (−1 phi), 0.85 mm (0.25 phi), 0.425 mm (1.25 phi), 0.25 mm (2 phi), 0.15 mm (2.75 phi), and 0.075 mm (3.75 phi).

### 2.3. Grid Sampling

At each site where we used this method (Figure 2), we established a grid as illustrated in Figure 3(d) by using a tape measure according to the procedure outlined by [14]. The length of the grid and width were 16 m and 7 m, respectively. Each pebble was randomly picked at 1 m interval, until total pebbles were 100. To ensure randomness in pebble selection, the sampler did not look at the riverbed while picking the pebble and practice was to draw each pebble from beneath toe-tip of the sampler's boot. Difficulties arise if the hand comes in contact with more than one particle. Reference [5] states that by selecting a specific point on the hand as a reference point (i.e., the right corner of the fingernail on the index finger) this problem can be overcome. We then measured the intermediate axis, grouped the pebbles into proper classes, and later plotted a frequency distribution. From the distribution, we also computed the distribution parameters as we did for volumetric samples.

## 3. Results and Discussion

### 3.1. Variation of Riverbed Material Using Volumetric Method

In the investigation of riverbed material, focus was on analysis of the changes induced by typhoons and torrential rains, which occurred between June and August 2012. In the mentioned period, there were three typhoons and their characteristics are given in Table 1.

**Figure 4:**
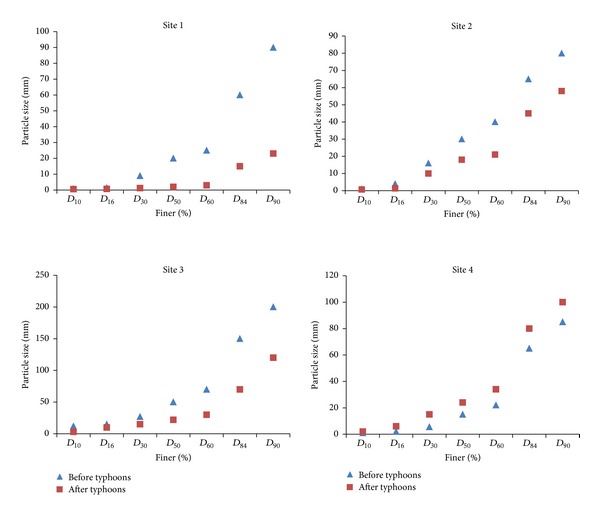
Grain size distribution before and after typhoon season using the volumetric method.

**Figure 5:**
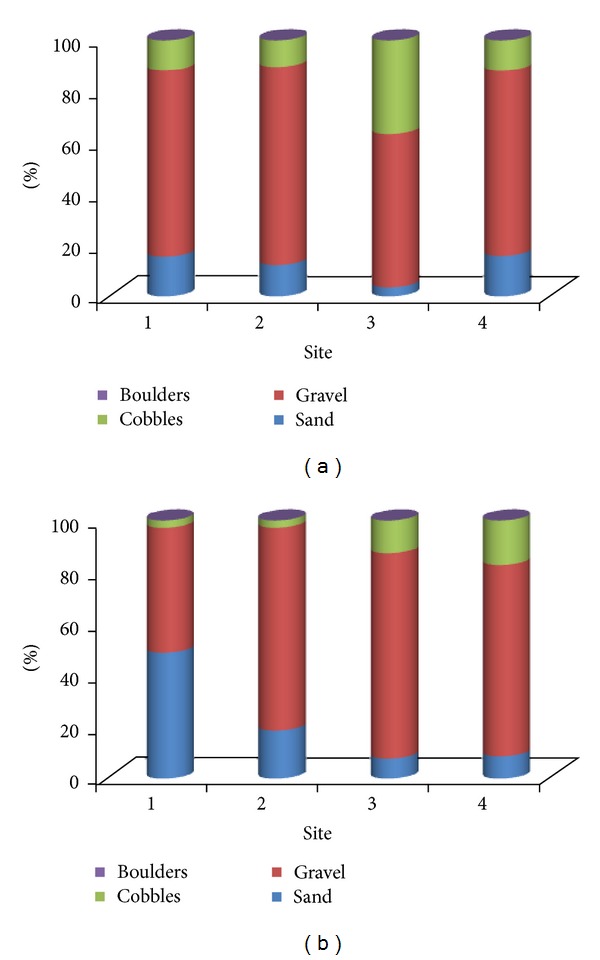
Proportions of clasts from volumetric method.

Figure 4 shows the distribution of grains prior to and after the typhoon season using the volumetric method. In all sites except site 4 there is a decrease in size for all sizes after typhoons. Site 4, which is the most upstream, showed an increase of grains sizes after typhoon. We attributed this to the fact that there are larger particles upstream since most of finer sediments are usually transported first. In site 1 (most downstream), there is a sharp decrease of grain size compared to other sites. For example, *D*_90_ is about 90 mm prior to and about 25 mm after typhoons. Site 1 is the closest to the sea; hence, the proportion of fine sediments increases. The average grain size (*D*_50_) show an increase from site 1 to site 4; *D*_50_ is about 2, 18, 22, 24 mm. However, the overall grain size after typhoons from site 1 to site 3 and site 4 shows a decrease when compared to before typhoon period (in order from site 1 to 4, *D*_50_ is 20, 30, 50, and 15 mm before typhoons, resp.). In site 3, the grain size is larger compared to other sites. This is because site 3 was closest to the bridge as shown by Figure 2. In addition, finer sediments transported from upstream as illustrated by Figure 5 raise the riverbed. Site 1 (downstream) before typhoon season is dominated by gravel class (2–64 mm) having 72.58% share and after typhoons the gravel class is reduced to 48.82%. The share of sand class (0.063–2 mm) increased from 15.60% to 48.82% after typhoons in site 1. There is a gradual increase after typhoons in the share of sand class from upstream in site 4 (48.82%) to downstream in site 1 (8.56%) and an increase in cobbles from site 1 (2.73%) to 17.15% in site 4.

### 3.2. Variation of Riverbed Material Using Grid Method

**Figure 6:**
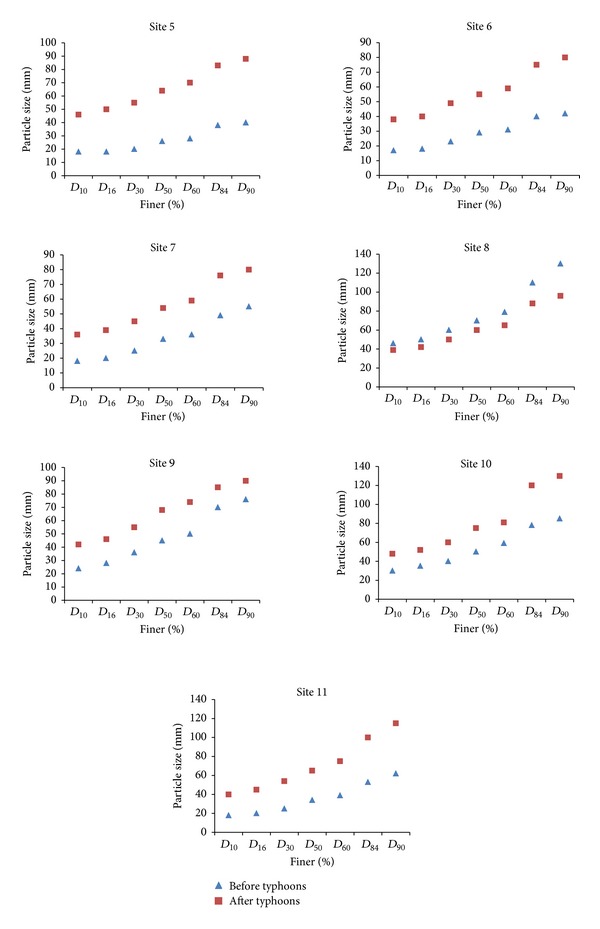
Grain size distribution before and after typhoon season using the grid method.

**Figure 7:**
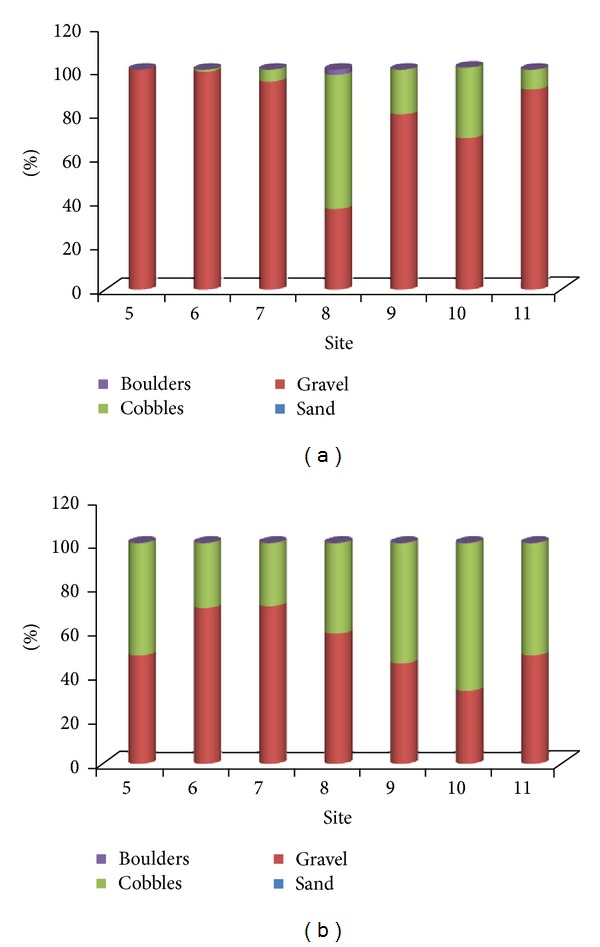
Proportions of clasts from grid method.

Figure 6 shows variability along Shi-Wen River using the grid method before and after the typhoon season. The variation of grain size observed with the grid is reverse compared to observations by volumetric method. There is an increase in grain sizes from all sites (5 to 11) except site 8. In this site, there is a reduction of grain size after typhoons. We assumed this was because of the tributary shown in Figure 2. Some of the finer sediments transported from this tributary were deposited in site 8. Before typhoons, there is an increase in bed material size between the two extreme sampling points. However, in between there is no regular pattern. After typhoons, the median grain size, *D*_50_, does not show much change as it is 64 mm downstream (site 5) and 65 mm upstream (site 11). *D*_90_ however, shows an increase from site 5 to site 8. Overall, there seems to be not much variation in all sites after typhoons, for example, *D*_50_ from site 5 to site 11 is 64, 55, 54, 60, 68, 75, and 65 mm, respectively.  Figure 7 demonstrate the proportion of the different clasts in each site. Site 5 (downstream), having 100% of gravel prior to typhoon season, shows a decrease in this class (49.11%) and an increase of cobble class (50.90%) after typhoon season. General observation in Figure 7 is the proportion of sand being halved except in site 8. In site 8, the share of gravel increases from 36.72% to 58.92% and cobbles are reduced from 60.94% to 41.07%. The share of boulders (greater than 256 mm) seen prior to typhoon season in site 8 is not observed after typhoon. Since the grid method involves picking up pebbles in the pavement layer, only coarser sediments will be available for picking, as floods will have removed the finer particles. This show that floodwaters transport grains selectively. Fine gravels are entrained first and displaced for the greater distances. The coarser gravels are mobilized only when the shear stress is large enough to entrain the clasts.

### 3.3. Comparison of Volumetric and Grid Methods

Table 2 shows the statistical parameters of the samples obtained from volumetric and grid methods. The Shi-Wen riverbed stream typically have values of median size (*D*_50_) of bed sediments exposed on the surface of 15 mm to 200 mm or larger (Figure 6). The subsurface sediments are usually finer by a factor of 1.5 to 3 [18]. The *S*_*g*_ of subsurface sediment size is typically larger in excess 3 being quite common. The geometric mean (*D*_*g*_ = (*D*_84_×*D*_16_)^0.5^) and the geometric standard deviation (*S*_*g*_ = (*D*_84_/*D*_16_)^0.5^) of the bed material prior to and after the observed season were computed and the results are shown in Table 2. *D*_84_ and *D*_16_ are the sizes below which 84% and 16% of the sample, respectively, are finer. The results are in line with those of [18] as the samples from volumetric method (site 1 to 4 in Table 2) had *S*_*g*_ in excess of 3 in both prior and after typhoon season indicating more variability within the sample. Moreover, the grid method (site 5 to 11 in Table 2) showed *S*_*g*_ of less than 1.6 indicating homogeneity of bed material on the surface. According to [19], a geometric standard deviation of less than 1.6 means the riverbed has uniform grain size distribution. The typhoon season does not show significant effect on the statistical parameters, since there is a slight reduction of the geometric coefficient after the season (Table 2). However, there is a clear difference with the geometric mean. After the typhoon season, for volumetric method there is a reduction while for grid method there is an increase, similar to observations in Figures 4 and 6, respectively.

### 3.4. Modality of the Grain Size Distribution

**Figure 8:**
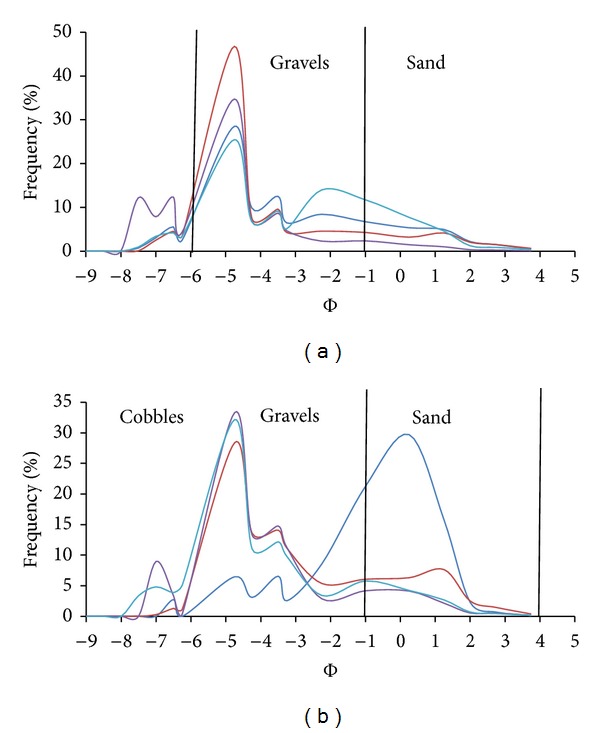
Histograms representing the grain size distributions for volumetric sampling (NB: *D* (mm) = 2^−Φ^).

**Figure 9:**
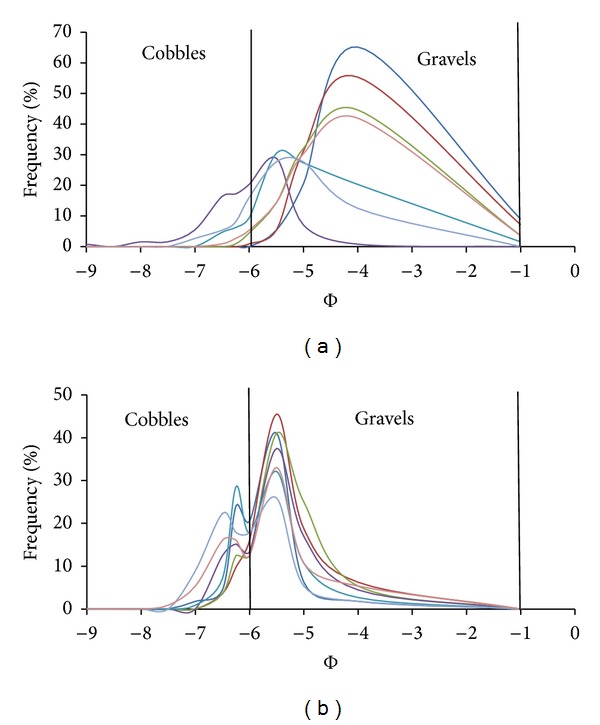
Histograms representing the grain size distributions for grid sampling (NB: *D* (mm) = 2^−Φ^).

A bimodal grain size distribution has two modes (distinct peaks in the frequency distribution), one in the finer and one in the coarser fraction. If the percent sand and fine gravels become high enough, the distribution becomes bimodal, developing a mode in the sand and in the gravel range. Bimodality can indicate the presence of two distinct particle size populations, supplied from a different source, with different abrasion resistance and each population may have had a different transport distance [10]. The recognition and characterization of the degree of bimodality are important for fluvial studies because incipient motion conditions differ in unimodal and bimodal sediment mixtures [15]. The degree of bimodality (*B*) for all the samples was calculated as outlined by (1) and the results are shown in Table 2. Our study did not emphasize the origin of modality; instead it focused on the possible changes in modality that maybe induced by floods. The modality grain size distribution was analysed separately for surface (grid) and subsurface samples (volumetric). Based on individual shares of cobbles, gravel, and sands that compose the river clasts, histogram distributions were calculated and are shown in Figures 8 and 9. From the figures, we can observe that bimodality is different from the surface and subsurface samples. In subsurface sample, the bimodality index is higher both before typhoons and after typhoons. Prior to typhoons, it ranges between 3.16 and 6.55, whereas after it ranges between 2.65 and 5.88. These indexes show that subsurface sediments are bimodal (*B* > 1.7) prior to and after typhoon season (Figure 8). Modes can be seen in the cobble class and gravel class prior to typhoon season. However, after typhoon, the samples show a polymodal (more than two modes) distribution because the proportion of sand increased showing another mode in the sand class.

The surface samples show a mode that is centred in the gravel class before typhoons (Figure 9(a)) and after a mode that is close to the cobble class (Figure 9(b)). The sediment layer exposed by the riverbed is usually washed of fine materials like sand; hence, only the coarser material will be left on the surface. Thus, grain distributions are unimodal (*B* < 1.7) as shown in Table 2 and illustrated by Figure 9. Reference [12] suggested that the main cause for bimodality in rivers is the lateral inputs of fine sediments. Our study reveals similar findings and further shows that typhoons accelerate the rates of bimodality.

### 3.5. Riverbed Material Transport and Bed Forms

**Figure 10:**
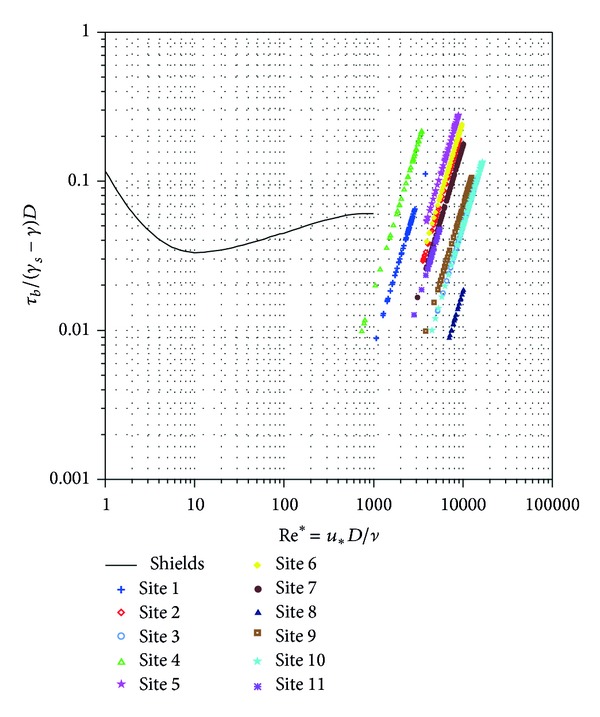
Shields diagram with data from all the sites using 10-year return period. (*τ*_*b*_/(*γ*_*s*_ − *γ*)*D*) in which, *τ*_*b*_ is bed shear stress, *γ*_*s*_ and *γ* are sediment and fluid densities, respectively, and *D* is the grain size to be moved multiplied by acceleration due to gravity. The parameter (*u*_∗_ *D*/*ν*) represents the Reynolds number, in which *u*_∗_ is the shear velocity and *ν* is the kinematic viscosity.

**Figure 11:**
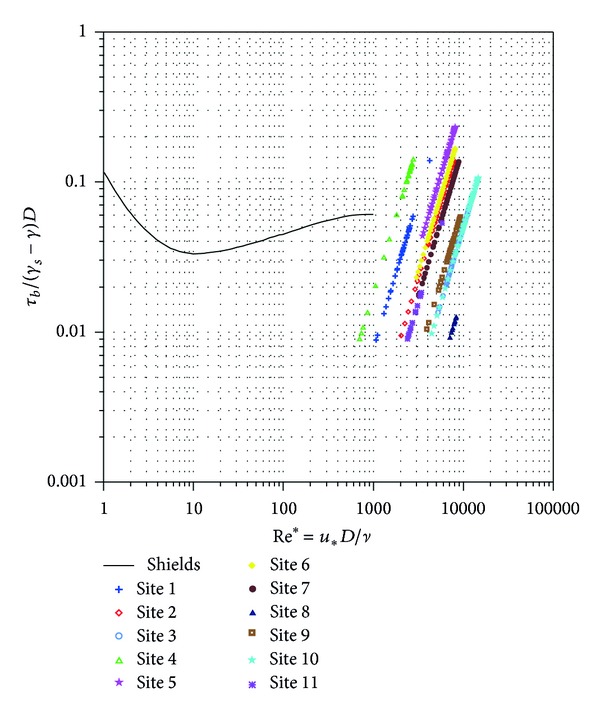
Shields diagram with data from all the sites using 2-year return period. (*τ*_*b*_/(*γ*_*s*_ − *γ*)*D*), in which *τ*_*b*_ is bed shear stress, *γ*_*s*_ and *γ* are sediment and fluid densities, respectively, and *D* is the grain size to be moved multiplied by acceleration due to gravity. The parameter (*u*_∗_ *D*/*ν*) represents the Reynolds number, in which *u*_∗_ is the shear velocity and *ν* is the kinematic viscosity.

From the above discussion and the change in grain size distribution, it is assumed that grains were selectively moved from upstream to downstream. Moreover, before any entrainment can occur, the flow must exceed a threshold of motion where the dynamic flow force exceeds the static force of the streambed. To sustain this assumption, the median grain size from all the sites investigated and the simulated flow conditions for the 10-year and the 2-year return period were used to plot on a Shields diagram in Figures 10 and 11, respectively. The Shields diagram represent the variation of the nondimensional threshold boundary shear stress (or threshold Shields parameter) with shear Reynolds number corresponding to the threshold of sediment entrainment [20]. It is considered the reference of any sediment transport research [21]. However, both figures showed that the points plotted were outside the range for Shields diagram. It was concluded that typhoon flows were high to best fit into this diagram, and these flow moved some grains from each site further downstream. Despite the wide application of Shields diagram, some researchers have expressed dissatisfaction [22–24] since the diagram less complies with the experimental data plots in the smooth and rough flow regimes. According to [25], Shields equation predicts sediment transport in fine or well-sorted sediments, while its application to poorly sorted sediments, typically of natural channels, has proven to be difficult.

**Figure 12:**
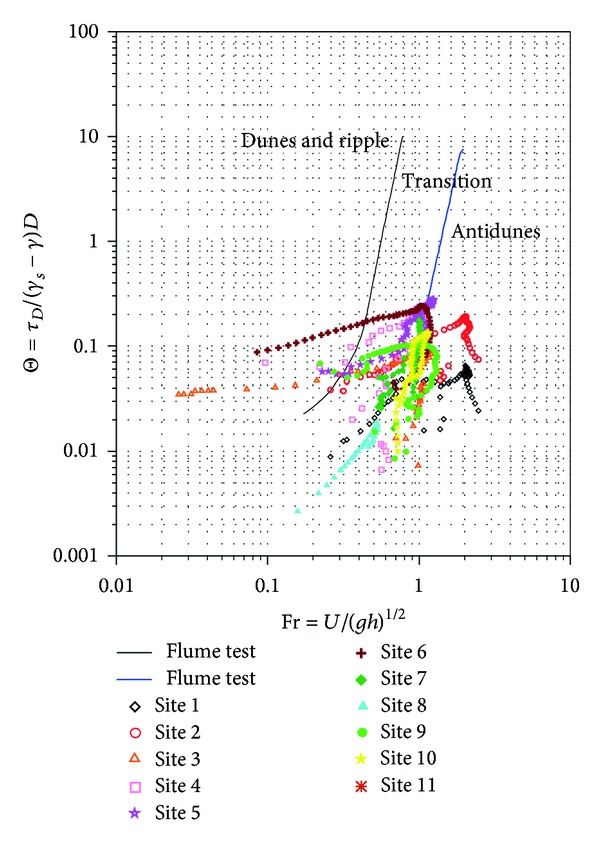
Shields number versus Froude number for Shi-Wen River using 10-year return period. (*τ*_*b*_/(*γ*_*s*_ − *γ*)*D*), in which *τ*_*b*_ is bed shear stress, *γ*_*s*_ and *γ* are sediment and fluid densities, respectively, and *D* is the grain size to be moved multiplied by acceleration due to gravity. The parameter ($U/\sqrt{gh}$) represents the Froude number, in which *U* is the flow velocity and *g* and *h* are the acceleration due to gravity and flow depth, respectively.

**Figure 13:**
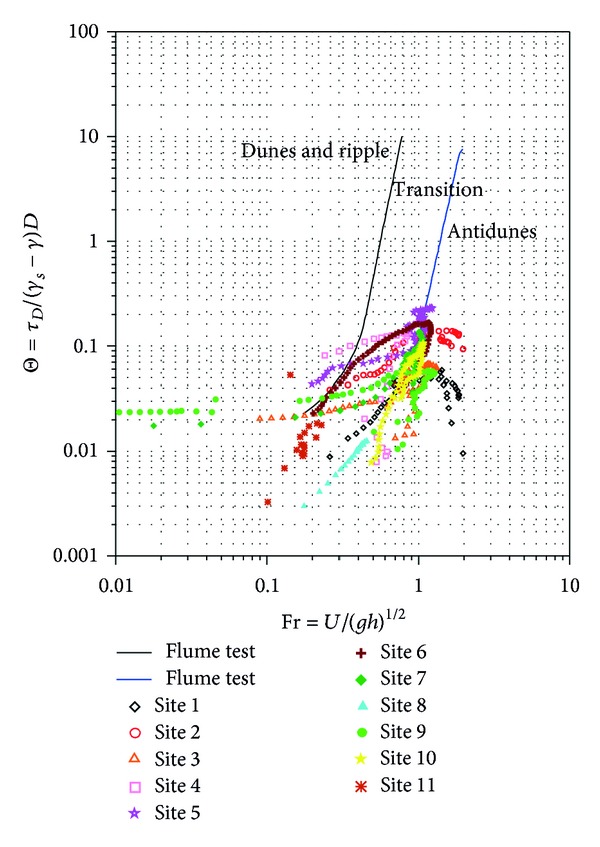
Shields number versus Froude number for Shi-Wen River using 2-year return period. (*τ*_*b*_/(*γ*_*s*_ − *γ*)*D*), in which *τ*_*b*_ is bed shear stress, *γ*_*s*_ and *γ* are sediment and fluid densities, respectively, and *D* is the grain size to be moved multiplied by acceleration due to gravity. The parameter ($U/\sqrt{gh}$) represents the Froude number, in which *U* is the flow velocity and *g* and *h* are the acceleration due to gravity and flow depth, respectively.

As a result of the storms, it is evident that characteristics of sediments in the riverbed changed, as illustrated in Figures 5 and 7. The high velocities generated during these events entrained some grains and deposited them further downstream, hence, altering and creating bed forms. It is generally agreed [12] that changes in river channels will take longer periods, but storms can accelerate those changes. Various bed forms are associated with various flow regimes. Therefore, the effects of storms in the creation of bed forms were estimated using 10-year and 2-year return periods and are shown in Figures 12 and 13, respectively. Reference [26] plotted the Shields number as a function of Froude number for the classification of bed forms in their flume experiments. Therefore, predictions of the resulting bed forms were based on their diagram. The Froude number is a dimensionless variable that describes the ratio of inertia to gravitational forces on flow [21]. It is a function of gravity and fluids average velocity and depth. When the Froude number is less than 1, gravity is the dominant force driving the fluids motion, and flow is subcritical. When it is greater than 1, inertia is the driving force; flow is called supercritical. Critical flow will occur when the inertial and gravitational forces are balanced and the Froude is 1.

In the 10-year return period, most of the Froude numbers are beyond 0.5 and less than 3, making most of the sites fall into the antidunes form, indicating that such floods could create antidunes in Shi-Wen River. The less flood flows (2-year return period) had Froude numbers concentrated between 0.2 and 1, and most of the sites fall on the transition form. In both return periods, only a few sites fell on the dunes and ripple form. The change in bed form reveals that Shi-Wen River's geometry and morphology is the direct consequence of the sediment transport process, in agreement with [25] when investigating bed material transport and morphology of alluvial river channels. In addition, the different bed forms will also influence the sediment transport in the river.

### 3.6. Roughness Coefficient

Roughness coefficient has an extensive effect on flow analysis of a river, including computation of velocity; hence, its estimation is crucial for design of hydraulic structures and stability assessment of revetments. In addition, it is also essential in the simulation of flow conditions associated with habitat suitability. Due to its importance and the significant relationship it has with riverbed material, we investigated the effects of typhoon season on its values. The roughness coefficient (*n*) based on empirical equations that relate the roughness coefficient to bed material was computed and results are shown in Table 3. In addition, to compute *n*, volumetric samples are used based on observation by [27]. They observed that surface grain size maybe more variable than subsurface samples and may require a large number of samples. These empirical formulas include the following:

 Strickler [28]: *n*_1_ = 0.047*D*_50_^1/6^,
 Einstein [29]: *n*_2_ = 0.0132*D*_65_^1/6^,
 Lane and Carlson [30]: *n*_3_ = 0.0256*D*_75_^1/6^,
 Meyer-Peter and Muller [31]: *n*_4_ = 0.038*D*_90_^1/6^.

The estimated *n* differs with the different methods; however, a common observation is that *n* is reduced after the typhoon period. Site 4, which is the furthest upstream, shows a smaller value of *n* before typhoon compared to sites 1, 2, and 3. After typhoon season, site 4 has the largest value of *n* compared to the other 3 sites. This is explained by the observation made in Section 3.3, which showed an increase in the share of cobble particles after the typhoon season and a reduction of gravel class due to erosion. Therefore, it is crucial to assess the values of *n* especially after such events as it is illustrated in Table 3. If, for example, the values of *n* prior to typhoon season are used for planning after the typhoon season, there will be an overestimation of *n*, resulting to an underestimate of flow velocity and an overestimate of river depth. Hence, engineering works will be greatly impacted.

## 4. Conclusion

Quantification of grain size distribution in riverbeds is an important issue in the study of river channel behaviour in hydraulic, geomorphology, and ecology. The changes in grain size distributions that may have been induced by typhoons and torrential rains have been investigated in this paper. These events have been shown to indeed induce some changes in the grain distribution. For volumetric samples, the share of sand class after the investigated season increased downstream (from 15.60% to 48.82%) and reduced from upstream (15.86% to 8.56%) indicating movement of sediment from upstream. Findings from the grid method shows that the 100% gravel observed downstream prior to typhoons is reduced to 49% after typhoons. The remaining 51% is occupied by cobbles. Further, torrential rains and typhoons can alter the bed form in a very short period to either antidunes or transition form, depending on their magnitudes in the study herein. The bimodality index had the same consistency with findings reported by other authors. Subsurface samples were found to be bimodal (*B* > 1.7) prior to typhoons and polymodal distributions are observed after typhoons. Moreover, surface samples were found to be unimodal. Prior to typhoons, the modal class is centred in the gravel class and after typhoons, the modal class shifts towards the cobble class. This showed that finer sediments including some gravels were moved during the typhoon season. Since there were great variations in the arrangement of grains after torrential rains, the roughness coefficient of the river was also altered. The empirical equations were employed, all showed a reduction in the roughness coefficient, indicating a necessity to consider such events in engineering works since they can alter roughness in a short time.

## Figures and Tables

**Table 1:** Hydraulic characteristics of the six torrential events.

Item	Event 4 (Typhoon Talim)	Event 5 (Typhoon Saola)	Event 6 (Typhoon Tembin)
Rainfall duration (h)	36	34	26
Rainfall amount (mm)	685	65	446
Peak *Q* (cms)	307	45	412
Max *Q*_*s*_ (Kg/s)	27675	6920	20652

**Table 2:** Statistical parameters of size distribution before and after typhoon season.

Site	Before typhoon season			After typhoon season		
	*D*_*g*_	*S*_*g*_	*B*	*D*_*g*_	*S*_*g*_	*B*
1	9.17	6.55	6.55	3.24	4.63	4.63
2	16.12	4.03	4.03	7.65	5.88	5.88
3	47.43	3.16	3.16	26.46	2.65	2.65
4	11.40	5.70	5.70	21.91	3.65	3.65
5	26.15	1.45	1.45	64.42	1.29	1.29
6	26.83	1.49	1.49	54.77	1.37	1.37
7	31.30	1.57	1.57	54.44	1.40	1.40
8	74.16	1.48	1.48	60.79	1.45	1.45
9	44.27	1.58	1.58	62.53	1.36	1.36
10	52.25	1.49	1.49	78.99	1.52	1.52
11	32.56	1.63	1.63	67.08	1.49	1.49

*D*_*g*_: geometric mean; *S*_*g*_: geometric standard deviation; *B*: Bimodality.

**Table 3:** Manning's roughness coefficient computed from empirical formulas.

Site	Before typhoon season				After typhoon season			
	*n*_1_	*n*_2_	*n*_3_	*n*_4_	*n*_1_	*n*_2_	*n*_3_	*n*_4_
1	0.077	0.023	0.048	0.080	0.053	0.016	0.035	0.064
2	0.083	0.025	0.050	0.079	0.076	0.022	0.045	0.075
3	0.090	0.027	0.057	0.092	0.079	0.024	0.049	0.084
4	0.074	0.023	0.048	0.080	0.080	0.024	0.051	0.082
